# Self-Reported Prevalence of Chronic Non-Communicable Diseases Concerning Socioeconomic and Educational Factors: Analysis of the PURE-Ecuador Cohort

**DOI:** 10.5334/gh.1416

**Published:** 2025-03-13

**Authors:** Camilo Felix, Mavel Lopez-Flecher, Michelle Vega, Katherine Andrango, Selena Andrango, Juan Marcos Parise-Vasco, Jaime Angamarca-Iguago, Daniel Simancas-Racines, Patricio Lopez-Jaramillo, Shrikant Bangdiwala, Sumathy Rangarajan, Salim Yusuf

**Affiliations:** 1 Grupo de Investigación en Enfermedades Crónicas no Transmisibles (CENIEC), Facultad de Ciencias de la Salud Eugenio Espejo, Universidad UTE, Quito, Ecuador; 2 Centro de Investigación en Salud Pública y Epidemiología Clínica (CISPEC), Facultad de Ciencias de la Salud Eugenio Espejo, Universidad UTE, Quito, Ecuador; 3 Instituto de Investigaciones Masira, Universidad de Santander (UDES), Bucaramanga, Colombia; 4 Population Health Research Institute, McMaster University and Hamilton Health Sciences, Hamilton, ON, Canada

**Keywords:** Non-communicable diseases, hypertension, diabetes mellitus, prevalence, poverty, sustainable development, Ecuador

## Abstract

**Background:**

The changing epidemiological landscape, marked by the increasing prominence of Non-Communicable Chronic Diseases (NCDs), underscores the need for studies that identify and analyze these conditions and their associated risk factors. This secondary analysis aims to describe the association between socioeconomic and educational characteristics and the prevalence of self-reported NCDs among participants in the PURE-Ecuador cohort in urban and rural populations of the Metropolitan District of Quito (MDQ), Ecuador.

**Methods:**

This secondary analysis is part of the Prospective Urban Rural Epidemiological (PURE) study. Data were collected from February to December 2018, including 2028 participants aged 35 to 70 years from different urban and rural areas of the MDQ. Data collection utilized standardized questionnaires administered in face-to-face interviews. Pearson’s chi-square tests and multivariate logistic regression were used to assess associations.

**Results:**

The self-reported prevalence of hypertension was 16.2%, rising to 32.7% in individuals over 60 years old. The prevalence of diabetes mellitus was 6.7%, coronary heart disease 1.3%, stroke 1.6%, heart failure 1.3%, COPD 0.4%, asthma 1.3%, and cancer 1.9%. Multimorbidity affected 5.9% of participants, with the highest rates in obese and older individuals (≥60 years). Adherence to medications was high for hypertension and diabetes mellitus but varied substantially between communities.

**Conclusions:**

The secondary analysis revealed significant disparities in the prevalence and management of NCDs in MDQ. The prevalence of self-reported NCDs in Quito, Ecuador, is significantly associated with age and body mass index (BMI). Older individuals, particularly those over 60 years, and obese participants demonstrated higher rates of NCDs and multimorbidity. While socioeconomic factors such as education and income showed some associations with NCD prevalence, these were less pronounced after adjusting for other variables. These findings highlight the importance of age-specific and obesity-focused interventions in addressing the burden of NCDs in this population.

## Introduction

The changing epidemiological landscape, characterized by the increasing importance of Non-Communicable Chronic Diseases (NCDs), highlights the need for studies that identify and analyze these diseases and their associated risk factors (1). The 2012–2013 Ecuadorian National Health and Nutrition Survey reported a high prevalence of hypertension and diabetes among adults over 17 years of age. According to the World Health Organization (WHO), the high prevalence of NCDs in low- and middle-income countries, together with an estimated 80% mortality rate due to these diseases, is linked to population aging, economic growth, and the emergence of NCDs risk factors (2).

The mortality profile has changed significantly since the first half of the 20th century, shifting from a predominance of deaths from infectious diseases to chronic diseases (3). The PURE study has provided crucial insights into the relationship between socioeconomic and educational characteristics and the prevalence of NCDs in different regions (4).

This article describes the association between the socioeconomic and educational factors of the Ecuadorian population enrolled in the PURE study and the prevalence of self-reported chronic non-communicable diseases among participants.

## Methods

### Study design

This secondary analysis is part of the PURE study, a large-scale longitudinal observational study designed to assess the impact of socioeconomic factors, lifestyle, and dietary risk factors on NCDs (5). The data for this report were collected from February to December 2018 as part of the baseline data collection for this study in Ecuador.

### Population selection

**Figure 1:**
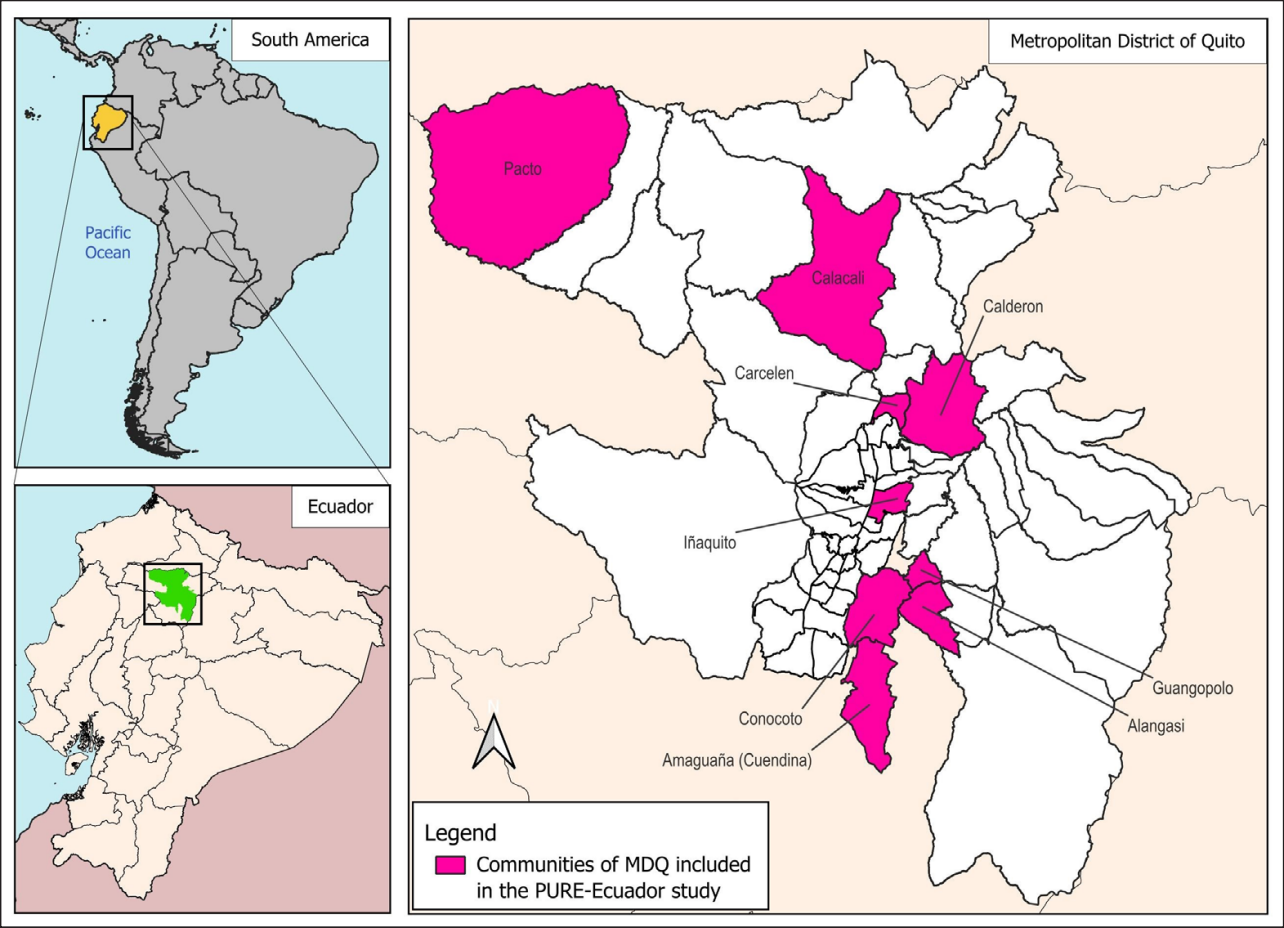
**South America map highlighting Ecuador and the Metropolitan District of Quito**. Map created by CISPEC, Universidad UTE using QGIS v3.34.7.

A total of 2028 participants aged 35 to 70 years were included. Participants were recruited from different urban and rural areas in MDQ (Figure 1). Individuals with a history of psychiatric disorders or mental retardation were excluded, as well as those with any condition that would prevent adequate understanding or completion of the data collection forms used in the study.

### Variables studied

The main variables of interest were the self-reported prevalence of several non-communicable diseases, including hypertension (HTN), diabetes mellitus (DM), asthma, coronary heart disease (angina, heart attack), stroke, heart failure (HF), chronic obstructive pulmonary disease (COPD), and cancer. In addition, it was collected data on socioeconomic factors such as educational level and household income, categorized according to the primary family food basket in Ecuador in 2018 (6).

### Data collection methods

Data were collected using standardized questionnaires administered in in-person interviews. These questionnaires included questions on medical history, lifestyle, and socioeconomic factors.

### Statistical analysis

Descriptive analyses were performed to estimate the prevalence of self-reported NCDs. Pearson’s chi-square tests and multivariable logistic regression analyses were used to investigate the associations between CVD and socioeconomic and educational factors. The models were adjusted for sex, age, level of education, marital status, income, and area of residence.

### Ethical considerations

The study was approved by the Human Research Ethics Committee of the Universidad San Francisco de Quito, under the code 2017–074E, issued on 21 July 2017. It was also approved by the Regulatory Authorities of the Ministry of Public Health of Ecuador, under code MSPCURI000221-4, granted on 26 October 2017. All participants provided their informed consent prior to enrollment.

## Results

### Relationship between self-reported chronic NCDs and socioeconomic and educational factors

A total of 2028 individuals aged 35 to 70 years were recruited in the PURE study in Ecuador. At baseline, participants had a mean age of 51.4 ± 9.8 years, 72.04% were women and 41.6% of the population belonged to rural areas. Table 1 presents the relationship between self-reported NCDs and sociodemographic factors. It details the prevalence of HTN, DM, CAD, stroke, HF, COPD, asthma, and cancer, considering sex, age, body mass index (BMI), education, marital status, household income, and area of residence.

**Table 1:** Relationship between self-reported chronic non-communicable diseases and sociodemographic factors in the Metropolitan District of Quito, Ecuador.


	SELF-REPORTED CHRONIC NON-COMMUNICABLE DISEASES															

	HTN		DM		CHD		STROKE		HF		COPD		ASTHMA		CANCER	

**Total (%)**	328	(16.2)	135	(6.7)	27	(1.3)	33	(1.6)	27	(1.3)	8	(0.4)	27	(1.3)	38	(1.9)

**95% CI**	(14.6–17.8)		(5.6–7.7)		(0.8–1.8)		(1.1–2.2)		(0.8–1.8)		(0.1–0.7)		(0.8–1.8)		(1.3–2.5)	

**Sex**																

Female (%)	241	(16.5)	96	(6.6)	17	(1.2)	26	(1.8)	23	(1.6)	6	(0.4)	20	(1.4)	34	(2.3)

Male (%)	87	(15.3)	39	(6.9)	10	(1.8)	7	(1.2)	4	(0.7)	2	(0.4)	7	(1.2)	4	(0.7)

**Age (years)**																

≤49 (%)	58	(6.4)	19	(2.1)	7	(0.8)	11	(1.2)	9	(1.0)	2	(0.2)	17	(1.9)	13	(1.4)

50–59 (%)	109	(17.2)	53	(8.4)	6	(1.0)	12	(1.9)	9	(1.4)	3	(0.5)	8	(1.3)	12	(1.9)

≥60 (%)	161	(32.7)	63	(12.8)	14	(2.9)	10	(2.0)	9	(1.8)	3	(0.6)	2	(0.4)	13	(2.6)

**Education (years)**																

None (%)	38	(30.7)	12	(9.7)	1	(0.8)	6	(4.8)	1	(0.8)	1	(0.8)	1	(0.8)	2	(1.6)

≤12 (%)	249	(16.1)	106	(6.8)	21	(1.4)	24	(1.5)	17	(1.1)	5	(1.2)	19	(1.2)	29	(1.9)

>12 (%)	41	(11.6)	17	(4.8)	5	(1.4)	3	(0.8)	9	(2.5)	2	(2.0)	7	(2.0)	7	(2.0)

**Marital status**																

Single (%)	30	(15.0)	11	(5.5)	2	(1.0)	1	(0.5)	2	(1.0)	2	(2.0)	4	(2.0)	3	(1.5)

Married/Free union (%)	234	(15.6)	92	(6.1)	18	(1.2)	25	(1.7)	16	(1.1)	5	(1.1)	16	(1.1)	27	(1.8)

Separated/Divorced/Widowed (%)	64	(19.5)	32	(9.7)	7	(2.1)	7	(2.1)	9	(2.7)	1	(2.1)	7	(2.1)	8	(2.4)

**Household income**																

<716 USD (%)	224	(16.7)	102	(7.6)	20	(1.5)	27	(2.0)	17	(1.3)	6	(0.5)	14	(1.0)	24	(1.8)

≥716 USD (%)	104	(15.2)	33	(4.8)	7	(1.0)	6	(0.9)	10	(1.5)	2	(0.3)	13	(1.9)	14	(2.1)

**Residence area**																

Rural (%)	125	(14.8)	59	(7.0)	8	(1.0)	18	(2.1)	7	(0.8)	5	(0.6)	5	(0.6)	13	(1.5)

Urban (%)	203	(17.1)	76	(6.4)	19	(1.6)	15	(1.3)	20	(1.7)	3	(0.3)	22	(1.9)	25	(2.1)

Body Mass Index (BMI)*																

Normal weight (%)	39	(8.7)	19	(4.3)	7	(1.6)	4	(0.9)	10	(2.2)	2	(0.5)	2	(0.5)	9	(2.0)

Overweight (%)	132	(14.4)	64	(7.0)	11	(1.2)	13	(1.4)	10	(1.1)	2	(0.2)	7	(0.8)	14	(1.5)

Obesity (%)	157	(23.8)	52	(7.9)	9	(1.4)	15	(2.3)	7	(1.1)	4	(0.6)	18	(2.7)	13	(2.0)


HTN: hypertension; DM: mellitus diabetes; CHD: coronary heart disease; HF: heart failure; COPD: chronic obstructive pulmonary disease. *Weight Normal: BMI 18.5 ≥ and < 25.0 kg/m^2^; Overweight: BMI 25.0 ≥ and < 30.0 kg/m^2^; Obesity: BMI ≥ 30.0 kg/m^2^.

The overall prevalence of HTN was 16.2% (95% CI, 14.6–17.8). Males exhibited a prevalence of 15.3%, while females reported 16.5%. The prevalence increased significantly with age, reaching 32.7% in individuals over 60 years old. Participants without formal education had the highest prevalence (30.7%), whereas those with more than 12 years of education had the lowest prevalence (11.6%). Individuals with monthly incomes below USD 716/month demonstrated a higher prevalence of HTN (16.7%) compared to those above this threshold (15.2%). Regarding BMI, the prevalence of HTN was 23.8% among obese individuals, compared to 8.7% among those with normal weight.

The prevalence of DM was 6.7% (95% CI, 5.6–7.7), slightly higher in males (6.9%) than females (6.6%). The prevalence of DM increased with age, reaching 12.8% in those over 60 years old. Participants without formal education showed a prevalence of 9.7%, while those with more than 12 years of education had a prevalence of 4.8%. Individuals with lower incomes reported a higher prevalence of DM (7.6%). Regarding BMI, the prevalence of DM was 7.9% among obese individuals versus 4.3% among those with normal weight (Table 1).

### Relationship between self-reported chronic NCDs multimorbidity and socioeconomic and educational factors

Overall, 74.3% (95% CI, 72.3–76.1) of participants reported no non-communicable diseases (NCDs), 19.8% (95% CI, 18.1–21.6) reported one disease, and 5.9% (95% CI, 5.0–7.0) reported two or more chronic diseases. No significant differences were found in NCD prevalence by gender (73.3% in females vs. 76.9% in males; p = 0.239) or area of residence (73.0% in rural areas vs. 76.0% in urban areas; p = 0.301). However, a significant difference was observed by age: 85.5% of individuals under 49 years reported no diseases, compared to 56.5% in those over 60 years (p < 0.001). Multimorbidity was more common among those with lower education levels: 8.9% of participants without formal education reported two or more diseases, compared to 6.2% of those with over 12 years of education (p < 0.001). Obese participants also showed a significantly higher prevalence of multimorbidity (8.7%) compared to those with normal weight (4.5%) (p < 0.001) (Table 2).

**Table 2:** Relationship between the number of self-reported chronic non-communicable diseases and sociodemographic factors in the Metropolitan District of Quito, Ecuador.


	NUMBER OF SELF-REPORTED CHRONIC NON-COMMUNICABLE DISEASES						

	0		1		≥2		P VALUE*

**Total (%)**	1506	(74.3)	402	(19.8)	120	(5.9)	

**95% CI**		(72.3–76.1)		(18.1–21.6)		(5.0–7.0)	

**Sex**							0.239

Female (%)	1070	(73.3)	301	(20.6)	90	(6.2)	

Male (%)	436	(76.9)	101	(17.8)	30	(5.3)	

**Age (years)**							<0.001

≤49 (%)	771	(85.5)	111	(12.3)	20	(2.2)	

50–59 (%)	457	(72.0)	135	(21.3)	42	(6.6)	

≥60 (%)	278	(56.5)	156	(31.7)	58	(11.8)	

**Education (years)**							0.001

None (%)	75	(60.5)	38	(30.7)	11	(8.9)	

≤12 (%)	1151	(74.3)	312	(20.1)	87	(5.6)	

>12 (%)	280	(79.1)	52	(14.7)	22	(6.2)	

**Marital status**							0.004

Single (%)	153	(76.5)	37	(18.5)	10	(5.0)	

Married/Free union (%)	1136	(75.8)	283	(18.9)	80	(5.3)	

Separated/Divorced/Widowed (%)	217	(66.0)	82	(24.9)	30	(9.1)	

**Household income**							0.012

<716 USD (%)	972	(72.3)	291	(21.7)	81	(6.0)	

≥716 USD (%)	534	(78.1)	111	(16.2)	39	(5.7)	

**Residence area**							0.301

Rural (%)	641	(73.0)	155	(18.4)	47	(5.6)	

Urban (%)	865	(76.0)	247	(20.8)	73	(6.2)	

**Body Mass Index (BMI)**							<0.001

Normal weight (%)	368	(82.5)	58	(13.0)	20	(4.5)	

Overweight (%)	698	(76.0)	177	(19.3)	43	(4.7)	

Obesity (%)	438	(66.5)	164	(24.9)	57	(8.7)	


*Pearson’s chi-squared test, p < 0.05.

### Relationship between chronic NCDs and the use of medications

The analysis of medication usage shows that 34.6% of respondents reported using medication in the past month. The total self-reported use of antihypertensive medication was 33.3%, while the use of antihypertensive medication among HTN was 86.4%. Furthermore, 14.1% of the participants reported using diabetes medications, and among those with DM, 84.3% reported using these medications. Among stroke patients, 17.6% reported using medication related to stroke. In the case of asthma, 7.1% of asthmatic patients reported using asthma medication, and 6.3% of those with CHD reported using cholesterol-lowering drugs.

#### Multivariate analysis of the number of self-reported chronic NCDs and sociodemographic factors

Multivariate analysis showed that age was the most significant factor for multimorbidity. Participants aged 50–59 years had a 3.08 times higher likelihood (95% CI, 1.78–5.34) of having two or more NCDs compared to those under 49 years, while those over 60 years had a 5.89 times higher likelihood (95% CI, 3.43–10.11). Education levels exhibited an inverse relationship with multimorbidity, though associations were not statistically significant after adjusting for other factors. BMI was also a significant predictor: obese individuals had a 1.87 times higher likelihood (95% CI, 1.09–3.21) of reporting two or more NCDs compared to those with normal weight (Table 3).

**Table 3:** Multivariate analysis of self-reported chronic non-communicable diseases and sociodemographic factors in the Metropolitan District of Quito, Ecuador.


	NUMBER OF SELF-REPORTED CHRONIC NON-COMMUNICABLE DISEASES							

	MODEL 1				MODEL 2			

	1		≥2		1		≥2	

**Sex**								

Male		Ref.		Ref.				

Female	0.84	(0.65–1.08)	1.17	(0.77–1.80)	1.07	(0.82–1.40)	1.08	(0.68–1.70)

**Age (years)**								

≤49 (%)		Ref.		Ref.				

50–59 (%)	1.93	(1.46–2.54)	3.13	(1.81–5.38)	1.80	(1.36–2.38)	3.08	(1.78–5.34)

≥60 (%)	3.31	(2.51–4.35)	5.89	(3.50–9.92)	2.98	(2.23–3.97)	5.89	(3.43–10.11)

**Education (years)**								

None (%)		Ref.		Ref.				

≤12 (%)	0.57	(0.38–0.85)	0.61	(0.32–1.18)	0.87	(0.57–1.33)	1.08	(0.54–2.13)

>12 (%)	0.39	(0.24–0.63)	0.68	(0.32–1.45)	0.75	(0.44–1.30)	1.59	(0.67–3.75)

**Marital status**								

Single (%)		Ref.		Ref.				

Married/Free union (%)	1.03	(0.70–1.50)	1.07	(0.54–2.10)	0.98	(0.66–1.45)	1.03	(0.52–2.06)

Separated/Divorced/Widowed (%)	1.46	(0.95–2.26)	1.91	(0.91–3.99)	1.17	(0.75–1.84)	1.50	(0.71–3.21)

**Household income**								

<716 USD (%)		Ref.		Ref.				

≥716 USD (%)	0.70	(0.55–0.89)	0.94	(0.64–1.39)	0.79	(0.60–1.04)	1.03	(0.65–1.64)

**Residence area**								

Rural (%)		Ref.		Ref.				

Urban (%)	1.17	(0.93–1.46)	1.11	(0.76–1.62)	1.24	(0.98–1.58)	0.97	(0.65–1.46)

**Body Mass Index**								

Normal weight		Ref.		Ref.				

Overweight	1.53	(1.11–2.09)	1.06	(0.62–1.82)	1.47	(1.06–2.03)	0.96	(0.55–1.67)

Obesity	2.12	(1.53–2.92)	2.04	(1.21–3.45)	1.93	(1.38–2.69)	1.87	(1.09–3.21)


Model 1: Crude OR (univariate). Model 2: Adjusted OR with sex, age, educational level, marital status, income, and location.

## Discussion

This secondary analysis identified the prevalence and sociodemographic factors associated with NCDS among participants in the PURE-Ecuador cohort in the MDQ. HTN and DM were found to be the most common NCDs in this study, with variations in the prevalence of asthma, CHD, stroke, COPD, and cancer among different communities, areas of residence, and age groups.

The self-reported prevalence of HTN was 16.2% in the entire study population, rising to 32.7% in those aged 60 years and over. This finding is consistent with the global PURE study, which analyzed socioeconomic status and cardiovascular disease risk in 20 low-, middle- and high-income countries and reported a high prevalence of NCDs in older adults. However, the global PURE study reported an overall prevalence of HTN of 32.4% and 43.4% in low- and middle-income countries, respectively (7). The prevalence in Ecuador differs from these findings and is similar to that reported in the Colombian PURE cohort (7). This variability may be because our and Colombian studies based their prevalence on self-reported HTN at baseline. In contrast, the global PURE study used diagnoses supplemented by physical examination and blood pressure measurement (7,8). This difference with the worldwide study may be due to differences in socioeconomic and educational characteristics in the included countries and geographical distribution.

In addition, obese people had a higher prevalence of HTN (23.8%) compared with normal-weight people (8.7%). These findings are consistent with previous studies showing that obesity is a risk factor for the development of HTN (9). The accumulation of adipose tissue contributes to endothelial dysfunction, peripheral vascular resistance, and activation of the renin-angiotensin-aldosterone system, mechanisms that increase activation of the renin-angiotensin-aldosterone system, which increases blood pressure (10).

In the Ecuadorian cohort, the overall prevalence of DM was 6.7%, with higher proportions observed in men and those without formal education. This finding highlights the possible influence of social and educational factors on the prevalence of DM, consistent with observations reported in the PURE study in Colombia, which had a DM prevalence of 5.7%, slightly lower than that reported in this study (7). However, in both populations, the prevalence of DM was higher in those with lower education and income levels. This suggests that education and socioeconomic status are critical social determinants of the increased prevalence of DM in both contexts. In addition, the study by López-Jaramillo et al. highlights that the prevalence of DM in rural and urban regions of Colombia may be influenced by dietary and lifestyle changes associated with socioeconomic development (11). This observation also applies to the Ecuadorian context, where differences in diet and lifestyle between urban and rural areas may contribute to differences in DM prevalence. These findings are consistent with previous studies indicating higher DM prevalence in populations with lower educational and socioeconomic levels (12,13,14). For example, the survey by Mente et al. on dietary factors and cardiovascular disease in the PURE cohort showed that diets poor in nutrients and high in refined carbohydrates are common in low-income populations, contributing to higher DM prevalence (14,15).

In addition, the prevalence of DM was higher in obese individuals (7.9%) compared to normal-weight individuals (4.3%), which is consistent with global studies indicating that obese individuals have a higher risk of developing diabetes compared to normal-weight individuals (16,17). These findings have important implications for public health policy and highlight the need for prevention strategies tailored to local contexts that promote healthy weight management.

The prevalence of CHD in the PURE-Ecuador cohort was 1.3%. This result is relatively low compared to the findings of Yusuf et al., who reported a higher prevalence of CVDs in low- and middle-income countries, especially in urban populations (4). The difference is mainly due to the small sample size in Ecuador (n = 2028) and possible variations in risk factors such as diet, access to health care, awareness of cardiovascular health, and differences in the implementation of primary and secondary prevention strategies.

The prevalence of stroke was 1.6%, with a higher prevalence in women and rural areas. These results are similar to those found in other PURE studies, where the prevalence of stroke was higher in women in several low- and middle-income countries (18). In addition, the high prevalence in rural areas may reflect limited access to medical care and differences in risk factors, such as uncontrolled hypertension. Studies by Yusuf et al. (4) and Gupta et al. (19) highlight the need for public health policies to improve equitable access to healthcare in rural areas.

In this study, the prevalence of NCDs multimorbidity increased significantly with age and decreased with higher levels of education. Similar trends have been observed in studies from Colombia and other countries in the region, where the prevalence of NCDs was higher among older people and those with lower levels of education (12,20,21). This highlights the influence of social determinants of health on the prevalence of multimorbidity and suggests that interventions should include strategies to improve access to health care for the most vulnerable groups.

Adherence to antihypertensive and diabetes medications was high among patients who self-reported these conditions, although access to drugs for other NCDs varied among the communities analyzed. This finding is consistent with the study by Gupta et al. in the PURE cohort, which showed that economic and educational factors influence disparities in access to medicines (19). Improving equitable access to essential medicines is critical for effective NCD management in Ecuador and other countries in the region. In other PURE studies, Chow et al. highlight the importance of the availability and affordability of essential medicines for preventing and managing NCDs (12,20,21).

In the case of diabetes mellitus, treatment adherence was high in most communities, averaging 84.3%. However, there were marked differences between some communities. For example, in Conocoto, adherence to diabetes treatment was 100%, while in Iñaquito, it was lower (66.7%). These results highlight the need to implement specific educational and support programs to improve treatment adherence in communities with lower compliance rates.

The low prevalence of medication use for asthma, cholesterol, and stroke in the entire sample is alarming. Only 0.3% of participants reported using asthma medications. This disparity suggests possible issues in the diagnosis and management of asthma, as well as a lack of access to essential medicines. Similarly, the use of medications for stroke (0.6%) and cholesterol reduction (6.3%) was deficient, indicating the underutilization of preventive treatments to avoid new cardiovascular and cerebrovascular events in the studied population. It has been shown that statins effectively control triglyceride levels and low-density lipoprotein cholesterol (LDL-C), which are common lipid abnormalities in the South American region (22,23) and are major risk factors for cardiovascular disease (24). Additionally, clinical practice guidelines recommend the use of statins as primary prevention for individuals with cardiovascular risk and secondary prevention in all patients who have suffered a cardiovascular event (23).

The observed differences in medication use among communities highlight the importance of considering local contexts and sociodemographic factors when planning health interventions. Urban communities like Iñaquito may benefit from interventions that optimize the use of existing resources, while rural communities like Guangopolo need strategies to improve access to the health system. Health policies should be tailored to address these disparities and ensure equitable NCD management across all areas.

In addition, the analysis of medication use among participants suggests that individuals may be using these treatments without having been formally diagnosed with hypertension or diabetes. This finding may be related to self-medication or empirical treatment based on non-specific symptoms, highlighting the need to improve diagnostic mechanisms and education on the appropriate use of medication.

The multivariate analysis showed that age is a determining factor in the prevalence of NCDs, particularly for those aged 60 years and over, who are significantly more likely to report two or more NCDs. This finding is consistent with previous studies showing increased multimorbidity with age (8,9,12,25,26). Additionally, although the relationship between education and multimorbidity was less pronounced in the adjusted models, the crude analysis suggested that lower educational attainment may be associated with a higher prevalence of multiple NCDs. These findings suggest that public health policies should prioritize education and support for older people to reduce the burden of NCDs.

The differences between crude and adjusted ORs may be due to the influence of adjustment variables. For example, age, BMI, and educational level can significantly impact the likelihood of reporting diseases; adjusting for these variables can change the observed relationship between sex and disease prevalence.

Age considerably impacts the likelihood of reporting self-reported diseases, both crude and adjusted. The adjusted results show a more significant relationship, indicating that older individuals are more likely to report diseases.

The small sample size of this secondary analysis, relative to the total population of Ecuador, limits the generalizability of these findings and makes direct comparisons with the PURE study in Colombia or other countries difficult. The observed differences in the low case numbers for specific pathologies, such as CHD, HF, asthma, and stroke, may be due to differences in diagnostic criteria, case definitions, or the demographic characteristics of the populations studied. It is essential to consider these potential sources of variation when interpreting the results.

## Conclusions

The PURE study has provided a comprehensive understanding of how socio-demographic factors influence the prevalence of NCDs in different global contexts. The present study revealed significant disparities in the prevalence and management of NCDs in the MDQ. Comparison of these findings with global PURE studies and the Colombian PURE cohort highlights the critical influences of socio-demographic and educational factors. These findings underscore the need for public health policies that address inequalities and improve access to education and health care to reduce the burden of NCDs.

## Additional File

The additional file for this article can be found as follows:

10.5334/gh.1416.s1 STROBE statement. STROBE Statement—Checklist of items that should be included in reports of ***cross-sectional studies***.

## Author Information

We confirm that this work is original and has not been published elsewhere nor is it currently under consideration for publication elsewhere. All authors have approved this study.
