# Supplementary material for: Self-Reported Prevalence of Chronic Non-Communicable Diseases Concerning Socioeconomic and Educational Factors: Analysis of the PURE-Ecuador Cohort
Source: Glob Heart. 2025 Mar 13;20(1):29. doi: 10.5334/gh.1416 (PMC11908430; doi:10.5334/gh.1416)
Supplement: STROBE statement. — STROBE Statement—Checklist of items that should be included in reports of cross-sectional studies. [file gh-20-1-1416-s1.pdf]

STROBE Statement—Checklist of items that should be included in reports of ***cross-sectional studies***

	Item No	Recommendation	Page No
**Title and abstract**	1	(*a*) Indicate the study's design with a commonly used term in the title or the abstract	Page 1; lines 1-3
		(*b*) Provide in the abstract an informative and balanced summary of what was done and what was found	Page 1, 2; lines 23-48
**Introduction**			
Background/rationale	2	Explain the scientific background and rationale for the investigation being reported	Page 3; lines 53-65
Objectives	3	State specific objectives, including any prespecified hypotheses	Page 3; lines 66-68
**Methods**			
Study design	4	Present key elements of study design early in the paper	Page 4; lines 70-74
Setting	5	Describe the setting, locations, and relevant dates, including periods of recruitment, exposure, follow-up, and data collection	Page 4; lines 70-80
Participants	6	(*a*) Give the eligibility criteria, and the sources and methods of selection of participants	Page 4; lines 76-80
Variables	7	Clearly define all outcomes, exposures, predictors, potential confounders, and effect modifiers. Give diagnostic criteria, if applicable	Page 4; lines 83-89
Data sources/ measurement	8*	For each variable of interest, give sources of data and details of methods of assessment (measurement). Describe comparability of assessment methods if there is more than one group	Page 4; lines 90-93
Bias	9	Describe any efforts to address potential sources of bias	N/A
Study size	10	Explain how the study size was arrived at	Page 4; lines 75-80
Quantitative variables	11	Explain how quantitative variables were handled in the analyses. If applicable, describe which groupings were chosen and why	Page 4; lines 83-89
Statistical methods	12	(*a*) Describe all statistical methods, including those used to control for confounding	Page 4; line 94-99
		(*b*) Describe any methods used to examine subgroups and interactions	N/A
		(*c*) Explain how missing data were addressed	N/A
		(*d*) If applicable, describe analytical methods taking account of sampling strategy	N/A
		(*e*) Describe any sensitivity analyses	N/A

Results

Participants	13*	(a) Report numbers of individuals at each stage of study—eg numbers potentially eligible, examined for eligibility, confirmed eligible, included in the study, completing follow-up, and analysed	Page 6; lines 110-111
		(b) Give reasons for non-participation at each stage	N/A
		(c) Consider use of a flow diagram	N/A
Descriptive data	14*	(a) Give characteristics of study participants (eg demographic, clinical, social) and information on exposures and potential confounders	Page 6; lines 110-132
		(b) Indicate number of participants with missing data for each variable of interest	N/A
Outcome data	15*	Report numbers of outcome events or summary measures	Page 6-7; lines 108, 109; 133, 134; 147; 156, 157
Main results	16	(*a*) Give unadjusted estimates and, if applicable, confounder-adjusted estimates and their precision (eg, 95% confidence interval). Make clear which confounders were adjusted for and why they were included	Page 7; lines 156-165; table 3
		(*b*) Report category boundaries when continuous variables were categorized	Table 1
		(*c*) If relevant, consider translating estimates of relative risk into absolute risk for a meaningful time period	N/A
Other analyses	17	Report other analyses done—eg analyses of subgroups and interactions, and sensitivity analyses	N/A
**Discussion**			
Key results	18	Summarise key results with reference to study objectives	Page 8-10; line 166-288
Limitations	19	Discuss limitations of the study, taking into account sources of potential bias or imprecision. Discuss both direction and magnitude of any potential bias	Page 14-15; line 242-252
Interpretation	20	Give a cautious overall interpretation of results considering objectives, limitations, multiplicity of analyses, results from similar studies, and other relevant evidence	Page 11; line 289-295
Generalisability	21	Discuss the generalisability (external validity) of the study results	Page 11; line 289-295
**Other information**			
Funding	22	Give the source of funding and the role of the funders for the present study and, if applicable, for the original study on which the present article is based	Page 11; line 305-307

*Give information separately for exposed and unexposed groups.

Note: An Explanation and Elaboration article discusses each checklist item and gives methodological background and published examples of transparent reporting. The STROBE checklist is best used in conjunction with this article (freely

available on the Web sites of PLoS Medicine at http://www.plosmedicine.org/, Annals of Internal Medicine at http://www.annals.org/, and Epidemiology at http://www.epidem.com/). Information on the STROBE Initiative is available at www.strobe-statement.org.
